# Interprofessional education in the integrated medical education and health care system: A content analysis

**Published:** 2016-07

**Authors:** MAHBOOBEH KHABAZ MAFINEJAD, SOLEIMAN AHMADY, SEYYED KAMRAN SOLTANI ARABSHAHI, SHOALEH BIGDELI

**Affiliations:** 1 Department of Medical Education, Faculty of Medicine, Tehran University of Medical Sciences, Tehran, Iran;; 2 Department of Medical Education, School of Medical Education, Shahid Beheshti University of Medical Sciences, Tehran, Iran;; 3 Center for Educational Research in Medical Sciences (CERMS), Department of Medical Education, Faculty of Medicine, Iran University of Medical Sciences, Tehran, Iran

**Keywords:** Interprofessional relations, Qualitative research, Medical education

## Abstract

**Introduction:**

The current literature supports the inclusion of inter-professional education in healthcare education. Changes in the structure and nature of the integrated medical education and healthcare system provide some opportunities for interprofessional education among various professions. This study is an attempt to determine the perceptions of students and faculty members about interprofessional education in the context of the medical education and healthcare system.

**Methods:**

This qualitative content analysis study was conducted using purposeful sampling in 2012. Thirteen semi-structured interviews were conducted with 6 faculty members and 7 students at Tehran and Iran Universities of Medical Sciences. Data collection and analysis were concurrent.

**Results:**

Data analysis revealed four categories and nine subcategories. The categories emerging from individual interviews were “educational structure”, “mediating factors”, “conceptual understanding”, and “professional identity”. These categories are explained using quotes derived from the data.

**Conclusion:**

Matching the existing educational context and structure with IPE through removing barriers and planning to prepare the required resources and facilities can solve numerous problems associated with implementation and design of inter-professional training programs in Iran.  In this way, promoting the development of a cooperative rather than a competitive learning and working atmosphere should be taken into account. The present findings will assist the managers and policy makers to consider IPE as a useful strategy in the integrated medical education and healthcare system.

## Introduction

Following the report of the World Health Organization (WHO) in 2010 entitled “Framework for Action on Inter-professional Education and Collaborative Practice”, educational planners were encouraged to move towards incorporating inter-professional education (IPE) and collaborative practice in services they deliver (1). It seems clear that traditional and discipline specific teaching and learning approaches provide fewer opportunities for achieving such a goal (2, 3). For this reason, IPE has been supported by educational planners as a possible strategy for improving inter-professional practice and, as a result, there has been a growth in inter-professional initiatives of health care education (4, 5).

Inter-professional education (IPE), defined as occasions when two or more professions learn with, from and about one another to foster collaboration in practice (6). Prior studies have noted the importance of IPE in making sense that the best care would be delivered by a team (7). There are strong relationships between inter-professional education and subsequent abilities of the practitioners who collaborate (8). Oliveira et al. reported that inter-professional education had positive effects on inter-professional collaboration and cultural awareness of students (9). Hence, it is hoped that early introduction of IPE in the curriculum may reduce the tendency to stereotype professional groups and enable them to collaborate effectively to improve health outcomes (10, 11). Dhaliwal commented on the importance of IPE in the development and sustenance of inter-professional collaboration; he believed that positive outcomes of IPE will be achieved through integrating IPE into health education curricula (12).

The scientific secretary of the first national conference on the prevention of medical errors, Dr. Delfan, stated that conflict in rendering healthcare services is the main cause of medical errors, and the best way to prevent it is to coordinate activities of healthcare members (13). It indicates that more attention is required to prepare the programs for strengthening inter-professional collaboration among healthcare members in Iran (14).

The inter-professional education in Iran has been studied in terms of background, structure, and their facilitating and inhibiting factors (15); however, few qualitative studies have explored in depth how faculty members and students perceive IPE in the Iranian context. The results of Irajpour et al.’s study showed that learning akin to IPE is more widespread than previously assumed in Iran (14).

The aim of this study was to explore the perceptions and viewpoints of faculty members and students about IPE in an integrated system. The researchers believed that understanding the perceptions of faculty and students towards inter-professional education can provide useful information for educational planners to design and implement such a program in the integrated system. 

## Methods

In this qualitative study, content analysis was applied (16). Purposive sampling was performed to select the participants, including both faculty members who had teaching experience or educational activities for at least 5 years and students who had spent more than 3 terms in clinical settings. Also, to ensure representative sampling, we collected our data from faculty members, who had teaching experience with two professions in clinical settings or classes, and students who had experience of learning with other students even if in informal way. The integrated medical education and healthcare system provide more opportunities for different professions to learn to work together. Therefore, we purposefully recruited the students and faculty members in order to have maximum variation in sampling, with males and females of various ages and experiences, and different professional disciplines.

Accordingly, six faculty members (3 males and 3 females) and seven students (2 males and 5 females) of Tehran and Iran Universities of Medical Sciences participated in this interview. All participants received a copy of the interview guide and gave their informed consent prior to being interviewed. The interview guide consisted of general questions, and participants had the opportunity to express their perspectives in detail. For example, the faculty members were asked to describe their understanding of IPE and then the interview was continued by asking some specific questions such as “What is your understanding of interprofessional education?”, “How would you describe your role in such programs?”, and “What factors have assisted you in designing and implementing IPE?”. Initial questions for students were “Are you involved with the members of the interprofessional education?”, “How are you encouraged to actively participate in the shared educational programs?”, and “What factors contribute to this?”

Thirteen semi-structured interviews were conducted individually. The time and place were determined based on the participant's preference. The interviews lasted about 30-50 minutes, depending on the interaction between the participant and the interviewee. Data collection continued until no new data was obtained, so that in the last two interviews, the participants pointed to similar issues.  Conventional approach to content analysis was used to analyze the qualitative data (17). Meaningful units of data were recognized and coded with appropriate lables. These codes were clustered under the subcategories and categories according to their similarities and differences, by means of comparative analysis. The extracted codes, categories and subcategories were examined and revised by the researcher.

In this study, trustworthy findings were presented in terms of dependability and confirmability, credibility and transferability. Accordingly, to increase the credibility of data, we used expert participants using purposive sampling, continuous reviewing of the data along with data collection simultaneously, data analysis immediately after the interviews, and using the results of an interview to modify and revise the questions of subsequent interviews. In addition, the data were member checked by the participants. Also, due to the dependability of data in this study, the researchers tried to record all interviews and data very carefully and ensure their accuracy by sending the primary analysis with initial codes to some participants. Also, to increase the transferability, we tried to have maximum variation in sampling, to present a deep description of our findings and then to compare them with other studies done in different settings. 

**Ethical considerations**

The research proposal was approved by Iran University of Medical Sciences Research Committee. All the participants were firstly informed about the purpose of the study and assured of anonymity prior to their participation in the study. Afterwards, they signed the informed consent forms. Interviews were tape recorded after the participants’ permission and transcribed with a code determined by the participants. 

## Results

A total of 13 subjects participated in this study (Table 1). Data were classified into four categories of educational structure, professional identity, mediating factors, and perception of the concept. These categories are explained using quotes derived from the data. The summary of the categories and subcategories obtained from the findings are presented in Table 2.

**Table 1:** Demographic characteristics of the participants

	**Faculty members**	**Students**
**No.**	6	7
**Mean age**	53 (41-65)	22 (21-24)
**Sex**	Female: 3	Female: 5
	Male: 3	Male: 2
**Specialty**	Medicine: 2	Medicine: 2
	Dental: 1	Midwifery: 1
	Nursing: 2	Nursing: 2
	Hygiene: 1	Operating room: 1
		Physiotherapy: 1
**Teaching experience**	Between 5 to 10 years: 1	
	Between 10 to 20 years: 4	
	More than 20 years: 1	
**Clinical experience**		Between 3 to 5 terms: 2
		Between 5 to 8 years: 5

**Table 2:** Data analysis results

**Categories**	**Subcategories**	**Code**	**Meaning units**
Educational structure	Facilitating conditions	Closeness of educational and clinical environment	“I have attended several common clinical rounds and I really can remember whatever I saw and heard … professors taught us excellent, explained and demonstrated all the cases to us, he considered us the same as his students.”
		Different forms of IPE	“As you know in many parts of the world they have same programs in their different universities. This lets the different universities to set up and run a variety of IPE programs like shared round, workshop, etc. We have similar ones in our country.”
		Strengthen the sense of collaboration	“The collaboration of different professions would be better established… if they interact with each other during the training sessions.”
	Barrier conditions	High workload	“We must do different activities in clinical settings… treatment, education, consulting, etc.”
		Compression of programs	“When we decide to set up our own class schedule, we have many problems, without any doubt coordinating a joint training schedule for different disciplines is a complex task…”
		Centralization of programs	“There is a fact that since our educational system is centralized, there is not any defined position for interprofessional education in educational programs of various fields!”
		Resistance of departments	“To me, one of the challenges in IPE in Iran is the resistance of departments…, heads of departments and managers not only do not consider IPE as a constructive approach…”
Mediating factors	Cultural atmosphere	Educational climate	“Different disciplines should put aside their cultural and mental barriers, and to have an interactive and adaptive relationship to solve problems. Unfortunately we never had the integrated culture.”
	Social conditions	The position of the professions	“Unfortunately, in our country some fields are not valuated … unfortunately, they are not seen as they must be seen.”
	Economic issues	Required resources	"At the first step, providing facilities is the most fundamental elements to run these programs…”
		Budget	“Through offering more budgets to the coordinated universities, we can encourage them to cooperate more.”
Professional identity	Professional merit	Familiarity with roles and responsibilities	“This shared training makes various professions to know about their roles and position in team…”
		A sense of usefulness	“These shared trainings made me interested in my discipline and feel useful…”
	Professional competence	Interprofessional knowledge and skills	“When I am being trained with other professions… I have the opportunity to exchange ideas and experiences with other students.”
Conceptual understanding	Design and implementation of IPE	Familiarity with application of IPE	“A professor for IPE needs requiring knowledge and skills and the collaboration of other disciplines’ professors.”
	Outcomes of IPE	Understand the consequences	“There are many outcomes of IPE such as improving teamwork, collaboration, changing views of different professions etc., which should be known by educational planners.”

**Educational structure**

The concept of educational structure means the process, rules and regulations in an educational system. There were various facilitating and inibiting conditions in such a system which was frequently mentioned by the participants.

***- Facilitating condition***

According to the participants of this study, the closeness of educational and clinical environment in such an integrated system, the tendency to strengthen the sense of collaboration among different professions, the possibility of designing the interprofessional education in various forms, and the movement toward reforming the current curricula were listed as facilitating conditions in educational structure to implement IPE. The tendency to strengthen the sense of collaboration among different professions is more required because of the high level of interaction between educational and clinical settings in the integrated medical education and healthcare system that are mentioned as the facilitating factors in the implementation of these programs. One participant said that:

*“When we go to teaching hospitals for our clinical courses, our interactions with various professions are close… exciting experience!*”* (Male nursing professor)*

The following is the comment of one of the participants on the possibility of implementing the inter-professional education in various forms as an educational strategy:

*“I want to say that it could run both in the theoretical program and clinical practice, both in the internship and in practical environment but it should be simulated* *.”* *(Male dentist professor)*

*“Ward round is a good place to learn inter-professional skills; we learn how professionals communicate with patients and other professionals in a real situation.” (Female nursing student)*

***- Barrier condition***

Although there are good opportunities for observing and practicing collaboration in such a system, a number of students and faculty members noted several difficulties in this setting. According to the participants of this study, compression of training programs, high workload of students in clinical settings, centralization of the current educational programs and resistance of different specialized departments of inter-professional education were the factors that fueled difficulties to implement IPE in some professions. They stated the difficulties of adjusting their professional responsibilities and interference of their time with educational programs, so that the main priorities for the students in the wards are commitment to the patient and education, respectively.

*“* *It happens too often that we want to go to the morning report shared with other disciplines, but …. We have no time to attend since we have to tackle our patient’s problems, and then deal with our training.” (Female nursing student)*

The difficulty of setting the schedules arises not only from the compression of educational courses, but also from the problems associated with coordinating the curricula of different professions and the centralization of the current educational programs.**

*“Coordinating the shared learning schedule of different disciplines is too hard...”  (Female physiotherapy student)*

*"* *We* *have a* *centralized system now; they cannot plan interdisciplinary method of teaching.”* *(Male dentist professor)*

**Mediating factors**

Mediating factors refer to the cultural atmosphere, social conditions and economic issues that have affected the implementation of these programs in the integrated healthcare and medical system.

***- Economic issues***

One of the participants believed that independent budget of each college can be considered as an obstacle for the time being. This participant has also made a suggestion for providing funding for joint educational programs:

*"* *When* *we* *'re* *budgeting,* *by offering better budget to the coordinated universities, we can encourage them to cooperate more.* *”* *(Female public health professor)* **

In addition, providing adequate resources and necessary funding to train large numbers of learners from different professions has been reported as a major challenge in the implementation of these programs. One of the participants said: 

*"As a result, a small university has no facilities to deal with the problems.” (Female nursing professor)*

***- Cultural atmosphere***

In addition to the economic situation, the inappropriate culture of individualism and uni-professionalism and unconstructive, competitive cultural atmosphere between different professions has some negative effects on the current cultural condition. The problem is seen not only in universities but also in the clinical settings. One of the participants said:

*“Unfortunately, in our society, everyone just accepts himself! Everyone in the hospital says that I’m better than the others… how can they work with each other while they have such ideas?” (Male nursing professor)*

One of the students mentioned the role of teachers in this regard:

*“I think our teachers or residents lead us to feel far from medical students, because they have always believed in a gap between us and themselves.” (Female midwifery student)*

Most professors focus on teaching the students of their own profession. 

*“Our works are essentially different. Teaching is completely distinct as job differences. What’s done by a nurse is very different from that of a doctor… training is directly related to the work we are going to do.” (Male medical student)*

***- Social conditions***

Undoubtedly, social condition is one of the most influential factors. The society’s negative views towards some professions and valuing some specific professions have increased the gap among different professions.

*“Unfortunately, in our country some fields are not validated … unfortunately, they are not seen as they must be seen.” (Male nursing professor)*

One of the students referred to the negative view of a patient as a member of society:

*“Once I entered the labor and wanted to interact with the patient, she told me: “only my physician is able to do everything for me. Now, what do you want to do for me?!!” She didn’t like me to even come close to her!! (Female midwifery student)*

**Conceptual understanding**

This category means familiarity with the meaning, ways and outcomes of applying IPE in the daily practice. 

***- Design and implementation of IPE***

Faculties, administrators and educational planners are other educational inputs that, due to insufficient understanding of the interprofessional education concept and lack of familiarity with the positive outcomes perceived from its implementation, have turned to uni-professional curricula. Faculty members referred to the necessity of educational authorities being more familiarized with how to develop shared educational contents and how to implement them.

*“Current curricula should have the potential of implementing interprofessional education…. But there is a need that educational authorities, at executive level, be familiarized with the content of inter-professional education and its advantages, specially its intensity or motivation. (Female medical professor)*

***- Outcomes of IPE***

The faculties’ insufficient knowledge of how the inter-professional education program is designed and implemented, difficulties of preparing a shared educational content as well as lack of awareness about the outcomes of interprofessional skills have reduced the faculties’ incentive and tendency to implement these programs.

*“The positive outcomes of such an education are not well-known…, and of course so are the difficulties of preparing and implementing such programs. Well, all of these make us fail in this context...” (Male medical professor)*

**Professional identity**

Professional identity is stated as one’s professional self-concept based on the roles, beliefs, values and experiences. The tendency to engage in the inter-professional education depends on the maintenance and enhancement of the professional identity of various professions. 

***- Professional merit***

From the participants’ viewpoint, students would better understand their value and professional position in delivering effective services to patients if they could get familiar with their roles and responsibilities of other professions via attending the shared programs. Moreover, the influence of positive experiences resulting from the shared learning with other professions in creating motivation and interest in the trainees to learn inter-professional skills is such that it largely changes the trainees’ feeling about their professional merit. One of the students reported that:

*“These shared trainings made me interested in my discipline and made me feel useful…” (Male operating room student)*

*“… It makes you so happy when you see your discipline’s effectiveness in a team.” (Female midwifery Student)*

***- Professional competence***

However, inter-professional outcomes will be achieved only if inter-professional interaction in the educational process is taken into consideration. According to the participants of this study, the mere presence of the learners of various disciplines together may not be much more effective. Almost all students acknowledged that inter-professional education, even in its accidental and unstructured form, was very useful and productive in enhancing their interprofessional capabilities.**

*“The impact of interprofessional education keeps us together to use the others’ information.” (Female nursing student)*

## Discussion

The purpose of this study was to explore the faculty members and students’ understanding of inter-professional education in the integrated healthcare and medical education system. The findings showed that the current state of inter-professional education in such a system is influenced by four categories, including educational structure, mediating factors, professional identity and conceptual understanding. 

In the participants’ perspective, integration in the health and education sectors in the Ministry of Health and Medical Education (MHME) can provide some opportunities for inter-professional education among various professions. These results are consistent with those of Irajpour et al. (1999), indicatingthat learning akin to IPE is more widespread than previously assumed (14).

The existence of factors such as close relationship between service provider milieus and educational environments in the integrated medical education and healthcare system, the possibility to run different types of inter-professional education programs (for example, shared workshops, joint training rounds, CME, etc.), and the opportunities of observing and practicing collaboration in the universities affiliated with educational hospitals may help to facilitate the implementation of such programs in the current conditions. Moreover, difficulties of adjusting the schedules among various professions, high workload of students in clinical settings, centralization of educational programs, and lack of attention to inter-professional skills and capabilities in the current curricula are all considered as barriers to implementing inter-professional education. There are similarities between these findings and those described by other researchers (18, 19). Accordingly, researchers believe that coordination of inter-professional experiences may require significant changes in the structure of curriculum in colleges. So, the deans, curriculum committees, and educational administrators must support these activities to reform the curriculum.

If we are to achieve effective inter-professional education, it seems reasonable that we consider the mediating factors. The results of the present study indicated some factors, including lack of sufficient budget and resources and the cultural and social conditions as barriers to such program. Thus, creating a context consistent with inter-professional education is an important issue referred to in almost all literature (20-22).

The participants of this research also mentioned the necessity of relative familiarity with the knowledge and outcomes of inter-professional education and emphasized the importance of this issue among different medical science professions which later provide services in the form of a team. In the researchers’ perspective, one common solution is to focus on selecting motivated professors and training them through faculty development courses. This finding is in agreement with Sterinert’s (2005) results which showed the negative attitude of faculties towards inter-professional education programs as a barrier to implementation, and considered the faculties’ development of courses a reactive solution to current challenges (23). In addition, the participants’ views indicated that communication and interaction with other professions as shared learning, especially in IPE will help to develop and improve the picture which students will hold of their professional identity in their mind. This finding corroborates the ideas of Lavender et al. (2014) who suggested that inter-professional education seems to provide an enhanced educational experience both in regard with the shared knowledge and in building a sense of communication and collaboration (24).

Generally, from the participants’ perspective, although the provision of shared educational programs such as IPE, even in informal form and as extra-curricular activities  in the current context has helped the students to better recognize different professional responsibilities of each other and to improve their communications, it can still be assumed that the most important characteristic of these programs, which is improving the interaction of students in different professions, has remained untouched. Further research on this topic needs to be undertaken. 

One of the limitations of this research was that the views of the managers and educational planners who are responsible for planning and implementation of curricula were not considered; this would certainly help to better recognize their views in order to design and offer inter-professional programs. Therefore, a study is recommended for further exploring of the views of the managers and educational planners. Also, enrolling the participants with different backgrounds and fields has some advantages according to the existing scientific documents (25); nevertheless, the chance to profoundly investigate different views of all professions was not made possible.

## Conclusion

The findings showed that a close relationship between medical education and healthcare system provides a more suitable condition for the transmission of values associated with IPE because the students observe and practice collaboration and interaction in a tangible manner. So, matching the existing educational context and structure with IPE through removing barriers and planning to prepare the required resources and facilities can solve numerous problems associated with implementation and design of inter-professional training programs in Iran. In this way, changing educational conditions by promoting the development of a cooperative rather than a competitive learning and working atmosphere should be considered. The present findings will help the managers and policy makers to consider IPE as a useful strategy in the integrated medical education and healthcare system. 
